# Case report of hepatic pseudocyst: A rare manifestation of liver metastasis from an anal squamous cell carcinoma

**DOI:** 10.1016/j.ijscr.2019.08.005

**Published:** 2019-08-17

**Authors:** Tomoaki Bekki, Yuji Takakura, Masatoshi Kochi, Kei Kushitani, Keiichi Mori, Koji Arihiro, Yoshifumi Teraoka, Hiroyuki Egi, Hideki Ohdan

**Affiliations:** a Department of Gastroenterological and Transplant Surgery, Applied Life Sciences, Institute of Biomedical and Health Sciences, Hiroshima University, Hiroshima, Japan; b Department of Pathology, Hiroshima University, Hiroshima, Japan; c Department of Surgery, Hiroshima Hiramatsu Hospital, Hiroshima, Japan

**Keywords:** SCC, squamous cell carcinoma, CT, computerized tomography, PET, Positron Emission Tomography, FDG, fludeoxyglucose, CRT, chemoradiotherapy, 5-FU, fluorouracil, Anal cancer, Squamous cell carcinoma, Liver metastasis, Pseudocyst

## Abstract

**Introduction:**

Liver metastasis of an anal squamous cell carcinoma (SCC) with a pseudocyst is uncommon. There are many diseases which form hepatic cystic lesions and sometimes it is difficult to arrive at an accurate diagnosis.

**Presentation of case:**

A 69-year-old woman visited our hospital with complaints of bloody stool and difficult defecation. A palpable mass in the anal canal was found on digital examination. The laboratory tests revealed anemia and raised levels of SCC antigen. On endoscopy a type 2 tumor was identified in the anal canal and biopsy revealed SCC. The patient was diagnosed with advanced anal SCC (cT2N1aM0, cStage IIIA) as a result of the examination and underwent chemoradiotherapy (CRT); unfortunately the tumor persisted after CRT. A salvage abdominoperineal resection with D2 and left lateral lymph node dissection was conducted. Histopathologically, the tumor was diagnosed as poorly differentiated SCC. She was readmitted for fever 3 weeks after surgery, and the abdominal CT showed multiple low density areas with enhancement at the edge of the liver, suggesting abscess or metastasis. The metastatic SCC was revealed by cytology of liver drainage and liver biopsy.

**Discussion:**

Anal cancers are sometimes encountered however; a pseudo cystic presentation of a liver metastasis is very rare which makes it difficult to distinguish from other diseases.

**Conclusion:**

We present a case of SCC in the anal canal with liver metastasis presenting as a hepatic pseudocyst.

## Highlights

• Liver metastasis of anal squamous cell carcinoma with a presented pseudocyst is very rare.
• Some hepatic diseases such as infections, simple biliary cysts, and neoplasms demonstrate a cystic change of the liver.
• Metastasis should be suspected when CT findings in patients with a history of gastrointestinal cancer show cystic changes involving the liver.

## 1 Introduction

Anal cancers are rare; comprising 2.5% of all gastrointestinal tumors and 0.4% of all new cancers [1]. The lung is the most frequent site for distant metastasis of squamous cell carcinoma (SCC), and liver metastasis occurs in 10% of SCC cases [2]. Additionally, hepatic neoplasms with cystic features are extremely rare [3]. Many cases indicate liver cystic change, such as infection diseases, simple biliary cysts up to cystadenomas and primary or metastatic malignancies. It is very difficult to arrive at an accurate diagnosis only by imaging techniques such as abdominal ultrasonography, abdominal contrast-enhanced computed tomography (CT), and magnetic resonance imaging. A detailed clinical history is important to confirm the diagnosis.

Metastatic SCC in the liver presenting as a hepatic cyst is extremely rare. We present a case of liver metastasis from a SCC in the anal canal with a formed pseudocyst. This work has been reported according to the SCARE criteria [4].

## 2 Case report

**Fig. 1:**
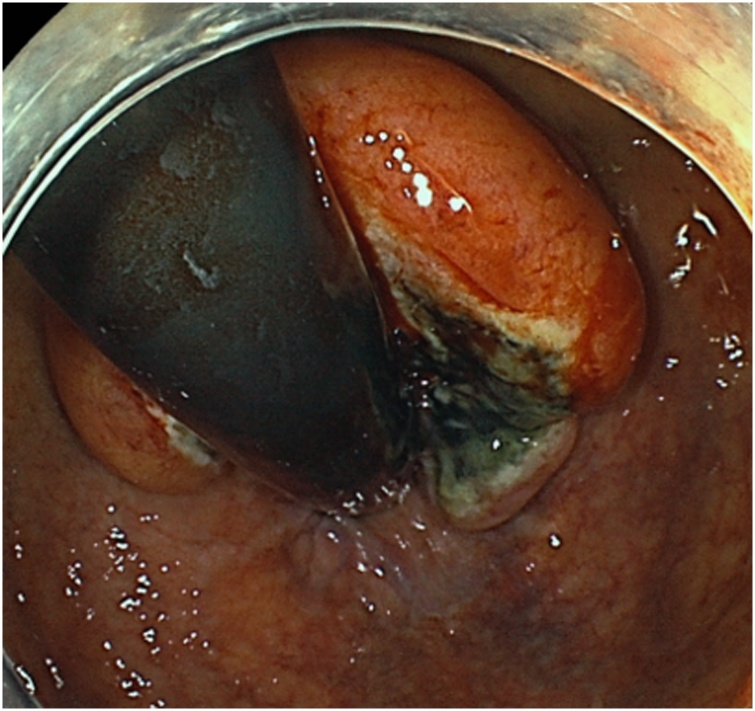
Endoscopic findings. Type 2 tumor found in the anal canal.

**Fig. 2:**
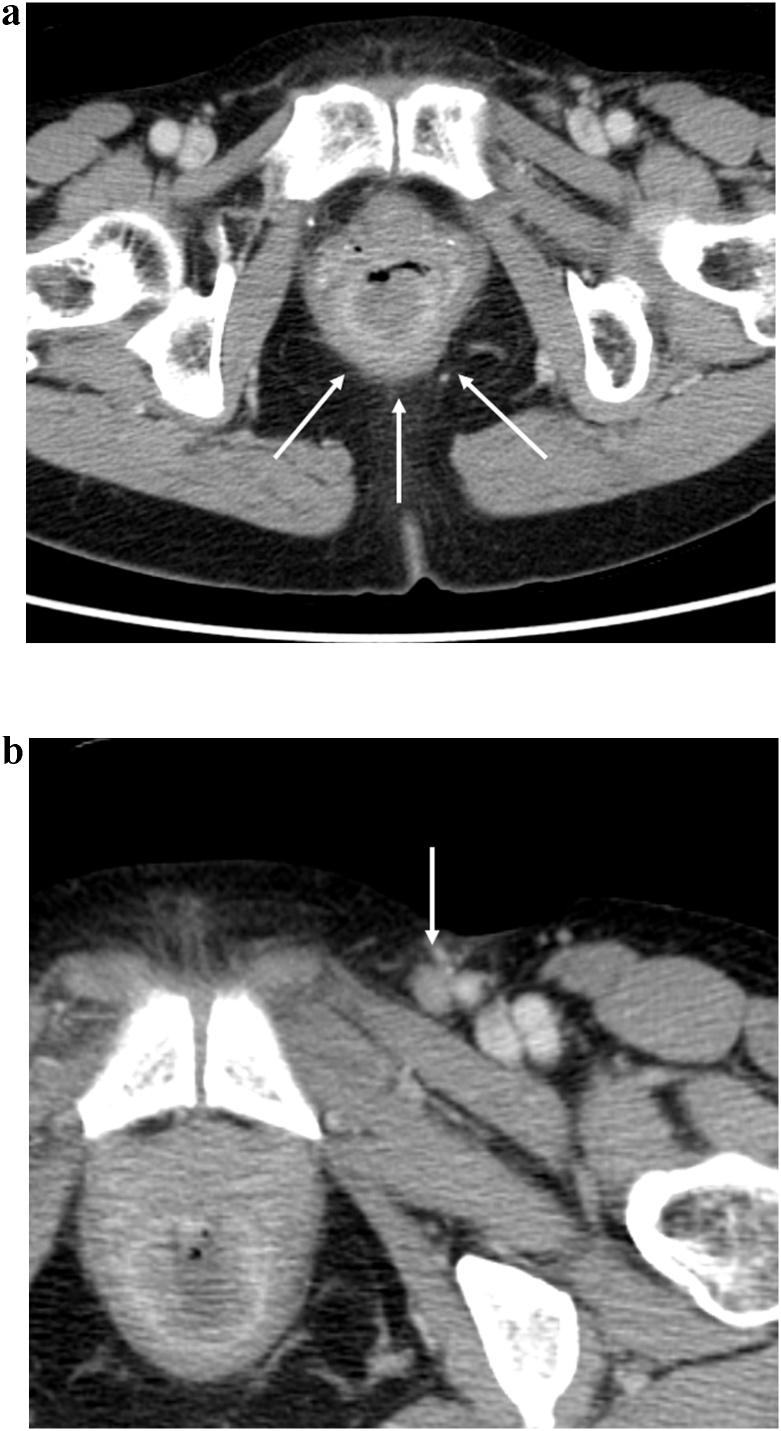
Abdominal contrast-enhanced CT findings. a: The mass with enhancement detected at the anal canal　(white arrow). b: An enlarged left inguinal lymph node (white arrow).

**Fig. 3:**
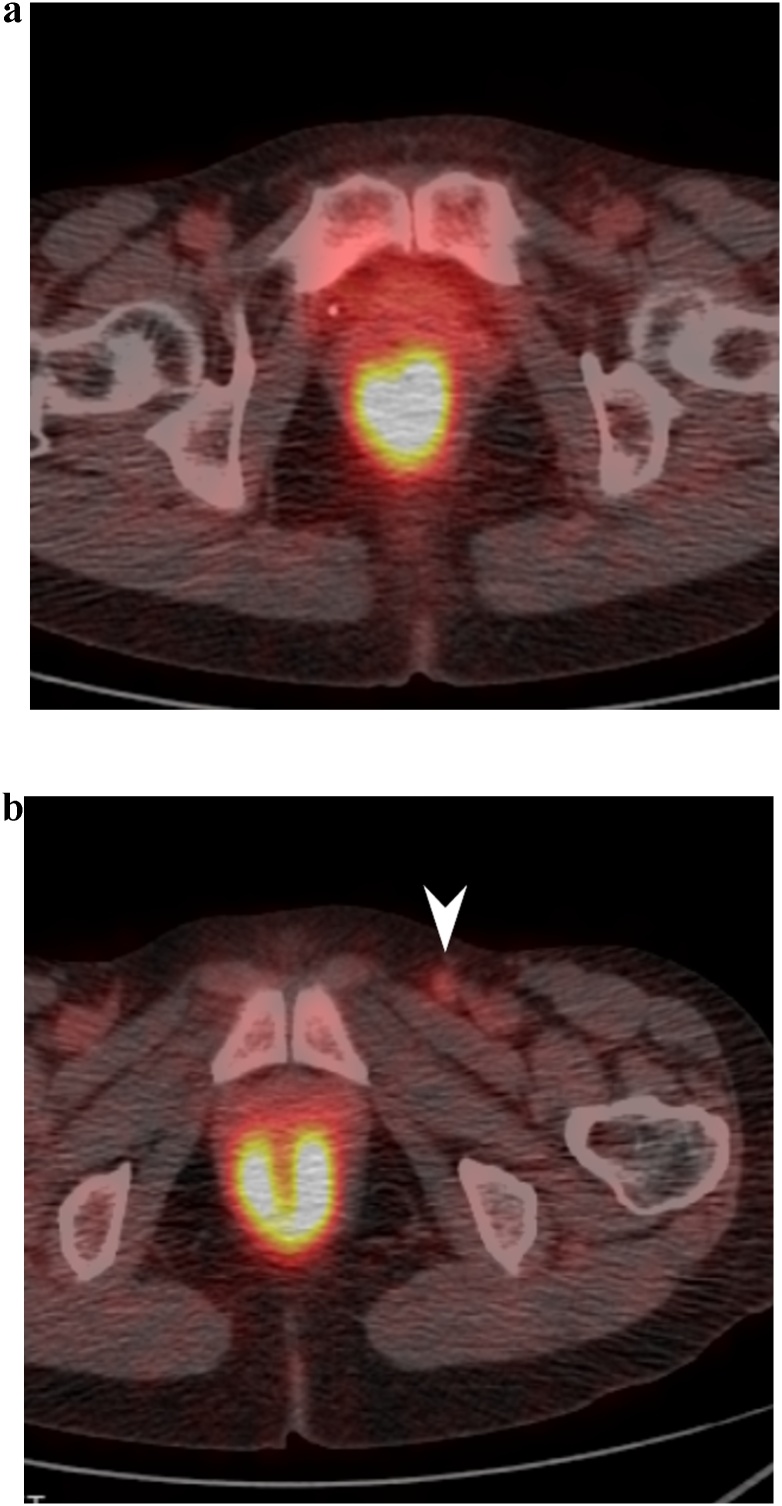
PET findings. a, b: PET revealed an accumulation of FDG in the anal canal mass and left inguinal lymph nodes (white arrow head) which were detected by CT.

**Fig. 4:**
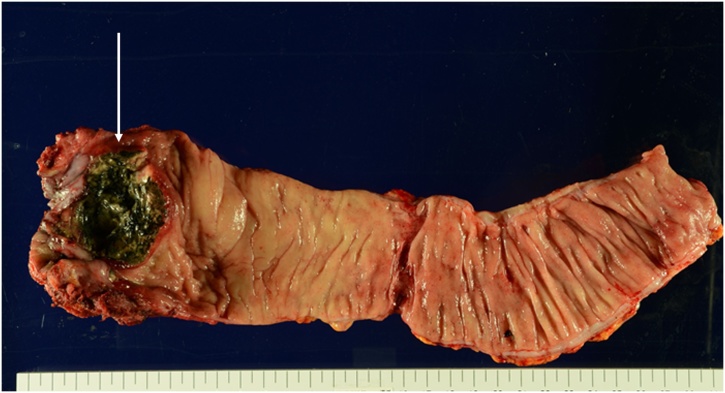
Macroscopic findings. Type 2 tumor encircling the wall found in the anal canal (white arrow).

**Fig. 5:**
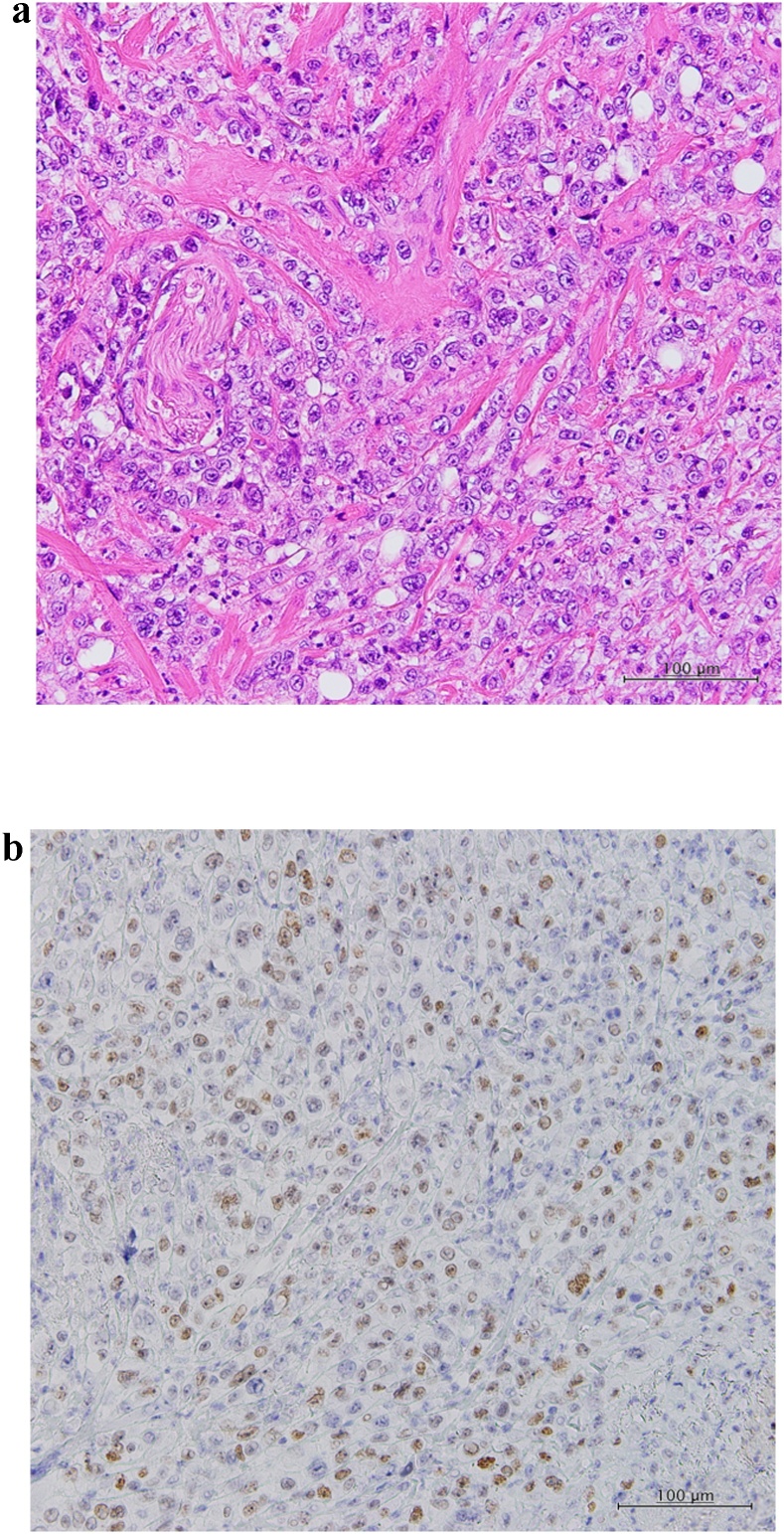
Histopathological findings. a: The tumor nuclei of different sizes and intercellular bridge led to a diagnosis of poorly differentiated squamous cell carcinoma (Hematoxylin-eosin stain, original magnification ×200). b: Tumor cells were positive for p63 (marker of basal cells) (original magnification ×200).

A 69-year-old woman was admitted to the Department of Surgery at our hospital for complaints of bloody stool and difficult defecation. She also had hypothyroidism, Sjogren’s syndrome, and uterine myoma. She was a non-smoker, did not drink alcohol, and had no history of surgery. A palpable mass was found in the anal canal on digital examination. The laboratory tests showed mild anemia, elevated levels of SCC antigen, and normal levels of serum carcinoembryonic antigen and carbohydrate antigen 19-9. On endoscopy, a type 2 lesion was found in the anal canal (Fig. 1), and on pathology, a diagnosis of SCC in the anal canal was made. The abdominal contrast-enhanced CT indicated that the mass was at the anal canal; swollen lymph node of left supraclavicular, left inguinal, and left external iliac lesions were also detected (Fig. 2a, b), and no evidence of liver and lung metastasis was noted. A Positron Emission Tomography (PET) scan revealed an accumulation of fludeoxyglucose (FDG) (11.5 F) in the anal canal and another mild accumulation of FDG (2.0 F) in the left inguinal lymph nodes (Fig. 3a, b). She was diagnosed with advanced anal SCC (cT2N1aM0 cStage IIIA) and was admitted to our hospital for chemoradiotherapy (CRT) with fluorouracil (5-FU) and mitomycin. An endoscopic examination after CRT revealed that the tumor had shrunk but persisted. Therefore, salvage laparoscopic abdominoperineal resection with D2 and left lateral lymph node dissection had to be performed for curative surgery. Macroscopically, a type 2 tumor was found all around the wall of the anal canal. (Fig. 4). Histopathologically, the tumor was diagnosed as poorly differentiated SCC. No metastasis in the regional and lateral lymph nodes was observed; however, massive venous invasion was detected. An immunohistochemical staining revealed that the tumor was positive for cytokeratin 5/6 and p63 (Fig. 5a, b). After CRT, the tumor was found to be Grade 1a on histopathology. The patient was diagnosed with anal SCC. The clinical course was uneventful and the patient was discharged on postoperative day 12.

**Fig. 6:**
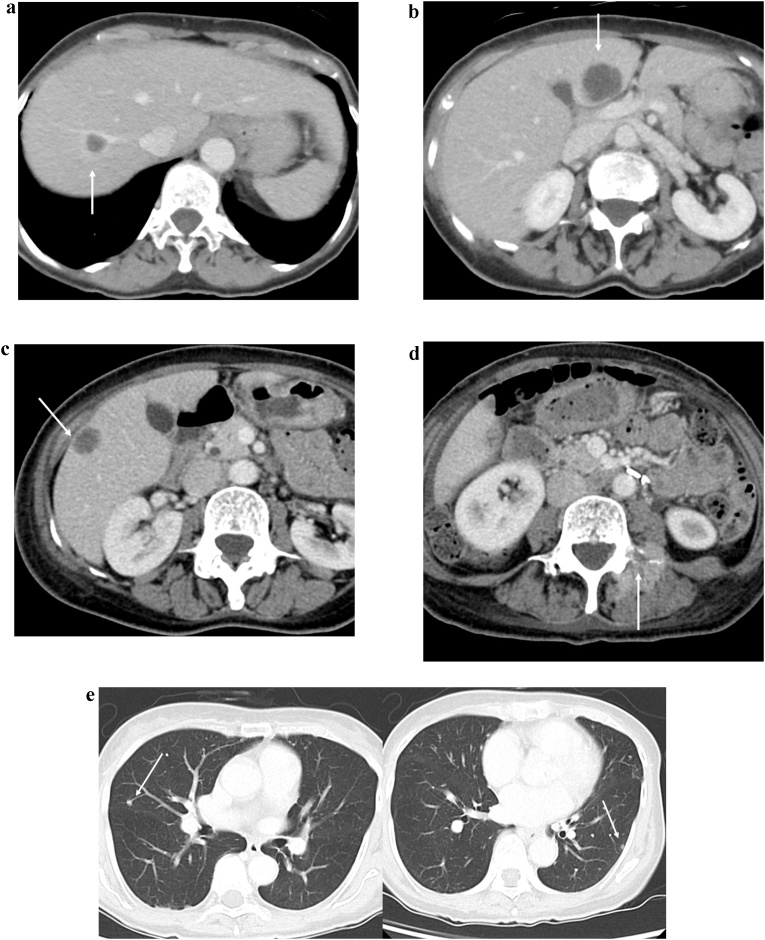
Abdominal contrast-enhanced computed CT findings 3 weeks after surgery. a–c: Few low-density areas with irregular peripheral rim enhancement in the liver. d: 3/4^th^ of lumbar spine melted. e: Multiple pulmonary nodules detected bilaterally (white arrow).

**Fig. 7:**
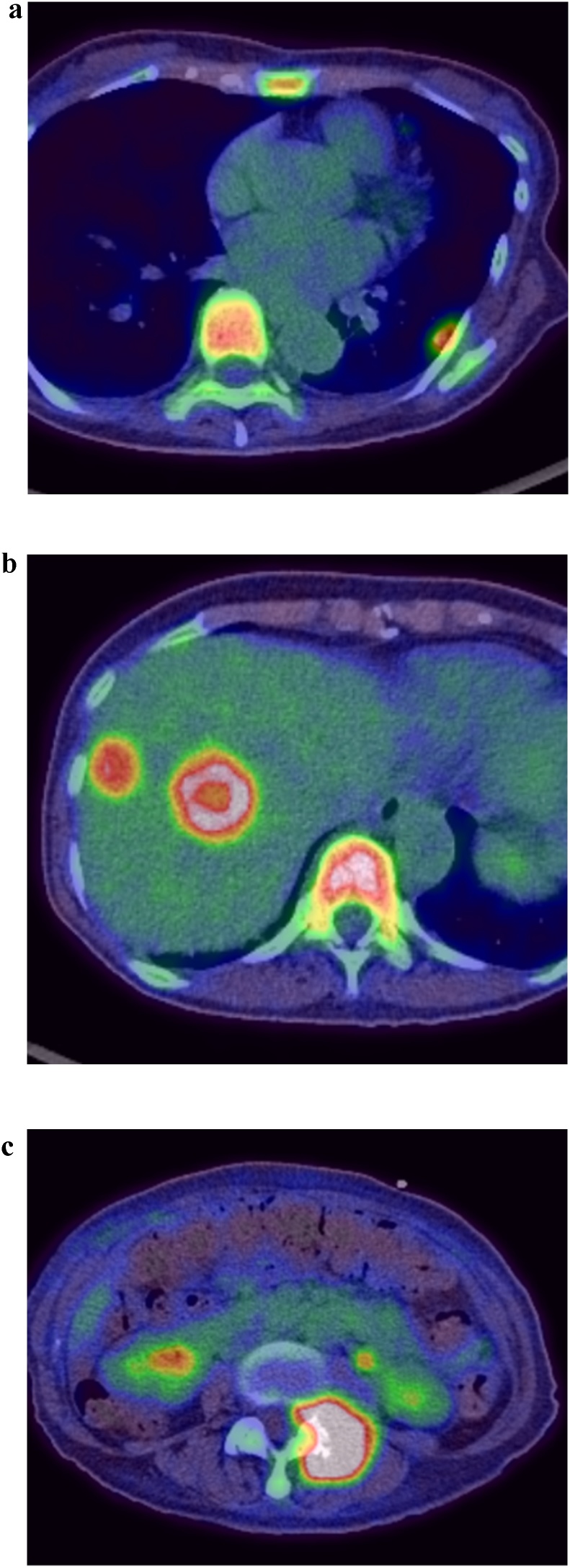
a–c PET findings after surgery. PET demonstrated an accumulation of FDG in the pelvic, lung, liver and vertebral body.

**Fig. 8:**
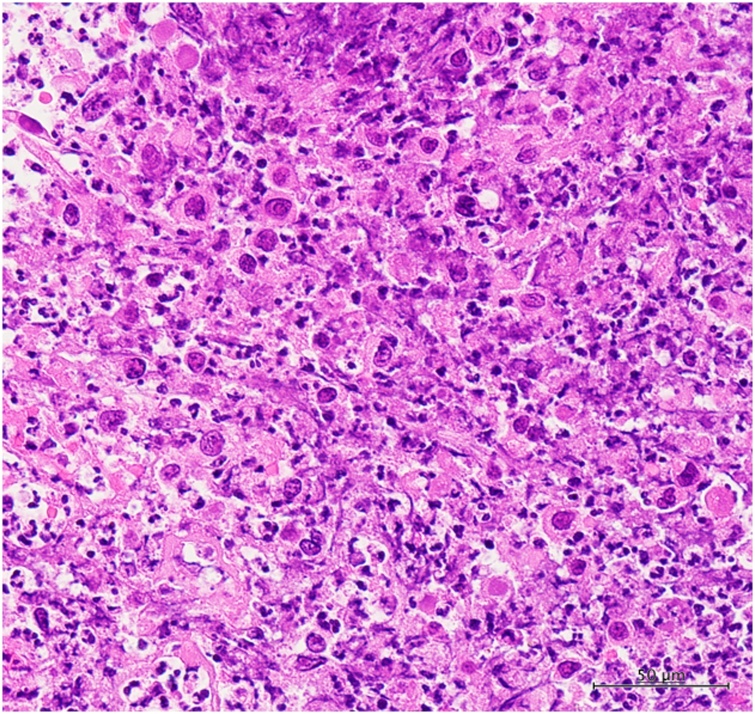
The liver biopsy findings. The liver biopsy necrosis similar to the tumor cells (Hematoxylin-eosin stain, original magnification ×400).

**Fig. 9:**
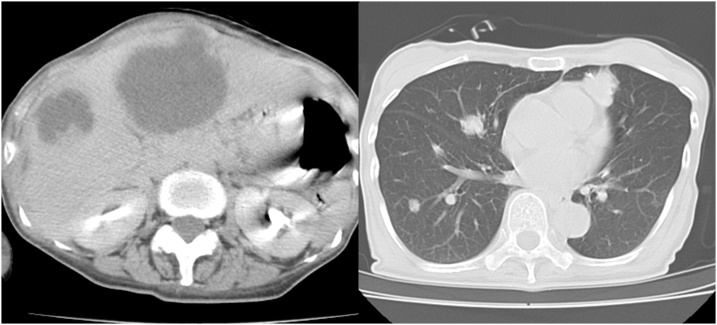
Abdominal contrast-enhanced CT findings 3 months after surgery Liver and lung metastases rapidly increased in size.

The patient was readmitted 8 days after discharge for fever, bilateral pedal edema, and perineal pain. White blood cell count and C-reactive protein levels were elevated. An abdominal contrast-enhanced CT showed low density areas in segment IV, V, and VII of the liver with enhancement at the edges. Multiple liver abscesses were initially suspected due to fever and elevation of inflammatory marker levels; however, whole body CT revealed melting of vertebral bodies, multiple lung nodule, and enlargement of the right inguinal lymph node (Fig. 6a–e), which suggested distant metastasis from anal SCC. Therefore, pseudohepatic manifestation of liver metastasis was suspected. PET-CT demonstrated an accumulation of FDG in the pelvis, lung, liver, vertebral body, and right inguinal lymph nodes (Fig. 7a–c). Percutaneous trans-hepatic abscess drainage was performed for diagnosis and treatment. A culture of the liver drain was negative, and fever continued despite the use of antibiotics. The cytology of liver drainage and liver biopsy revealed metastatic SCC with necrosis (Fig. 8). The patient was diagnosed with hepatic pseudocystic metastasis of anal SCC and underwent chemotherapy with 5-FU and cisplatin after palliative radiation to the vertebral body. Although she underwent chemotherapy for 2 courses, abdominal contrast-enhanced CT 3 month after surgery revealed liver and lung metastasis with increasing size (Fig. 9).

## 3 Discussion

Hepatic cysts are often encountered in clinical practice, occurring in 2.5% of the general population [5] with a higher proportion of them occurring in females (female: male ratio 1.5:1). Most hepatic cystic lesions are benign [6], however, it is important to distinguish benign cysts from harmful ones such as echinococcosis, amebic abscess, cystadenoma, and cystadenocarcinoma [7,8]. Additionally, there have been reports of liver metastases presenting with cystic change. Alsolaiman et al. reported the first case of metastatic SCC to the liver from the uterine cervix presenting as hepatic pseudocyst [9]. Federle et al. estimated that only 1% of hepatic neoplasms developed cystic changes [10]. A pseudocyst formed due to a liver metastasis has sometimes been reported as secondary to neuroendocrine tumors or ovarian malignancies [11,12]. Also, cystic changes are often reported in liver malignancies after chemotherapy because of the resulting induction necrosis [3]. A hepatic pseudocyst has no specific symptoms or specific imaging findings. Robinson PJ [13] showed that cystic liver metastases usually have an irregular peripheral rim of enhancement which was also seen in the present case. However, these CT findings are also found in cystic hepatocellular carcinoma and liver abscess which makes diagnosis difficult [14].

Borhani et al. [15] developed a simplified algorithm for identifying and differentiating cystic hepatic legions. When CT findings reveal solitary or multiple lesions, various differential diagnoses should be considered; such as infections, benign legions as simple biliary cysts, and primary or metastatic malignancies [15,16]. Metastatic lesions must be considered as part of the diagnosis if the patient has a history of extra hepatic malignancy; and pyogenic abscess if there are clinical signs of infection. In cases with CT findings of cirrhotic liver and hypervascular components, cystic hepatocellular carcinoma must be ruled out. Lantiga MA et al. [17] showed also developed an algorithm for diagnosis of hepatic cyst lesions. We should consider cystic neoplasm which showed vascular flow within septa on contrast-enhanced ultrasound and echinococcus antibodies were negative. A detailed clinical history is very important for confirmation of the diagnosis. The pathological findings from biopsy, cytology, and culture of aspirate from the cystic region are essential for accurate diagnosis [9,10,18]. If a CT shows evidence of a cystic liver with a positive history of gastrointestinal cancer, a pseudocyst from a liver metastasis should be considered as a diagnosis which should be confirmed by a biopsy of the cystic lesion.

The reasons for the hepatic cystic changes were unclear. The pathological findings of this case showed poorly differentiated SCC and massive venous invasion; recurrence after surgery was extremely early and the disease progression was very quick. We theorize that the poor pathological findings led to rapid tissue necrosis, which was revealed as a liver pseudocyst on the abdominal CT with poor response to chemotherapy.

We could not find any previous reports on liver metastasis from anal SCC forming a pseudocyst, and to the best of our knowledge, this is the first reported case.

## 4 Conclusions

We identified a rare case of liver metastasis from an anal squamous cell carcinoma with a formed pseudocyst. It is important arrive at a comprehensive diagnosis from the physical examination, imaging findings and detailed clinical history of patients.

## Sources of funding

This research received no specific grant from any funding agency in the public, commercial, or not-for-profit sectors.

## Ethical approval

E-477-3.

## Consent

Written informed consent was obtained from the patient for publication of this case report and any accompanying images.

## Author’s contribution

All authors in this manuscript contributed to the interpretation of data, and drafting and writing of this manuscript. Tomoaki Bekki is first author of this paper. Yuji Takakura is corresponding author of this paper. Tomoaki Bekki, Yuji Takakura, Masatoshi Kochi and Hiroyuki Egi conceived and designed the study and drafted the manuscript. Tomoaki Bekki, Yuji Takakura, Masatoshi Kochi, Yoshihumi Teraoka and Hiroyuki Egi were engaged in patient’s care in our hospital including surgery. Kei Kushitani, Keiichi Mori and Koji Arihiro diagnosed SCC.

Hideki Ohdan contributed to study concept, and review of the final manuscript and submission of the paper. All the authors read and approved the final manuscript

## Registration of research studies

The manuscript does not report the result of an experimental investigation or research on human subjects.

## Guarantor

Yuji Takakura.

## Provenance and peer review

Not commissioned, externally peer-reviewed.

## Declaration of Competing Interest

None of the authors have any commercial or financial involvement in connection with this study that represents or appears to represent any conflicts of interest.
